# Supplementary material for: Bacterial Nanocellulose from Komagataeibacter Medellinensis in Fique Juice for Activated Carbons Production and Its Application for Supercapacitor Electrodes
Source: Polymers (Basel). 2023 Apr 1;15(7):1760. doi: 10.3390/polym15071760 (PMC10096803; doi:10.3390/polym15071760)
Supplement: Supplementary file 1 [file polymers-15-01760-s001.zip › polymers-2262543-supplementary.pdf]

Supplementary Information for

Bacterial nanocellulose from *Komagataeibacter* ***Medellinensis*** in fique juice for activated carbons production and its application for supercapacitor electrodes

Juliana Villarreal-Rueda [1], Zulamita Zapata-Benabithe [2,*], Laia Posada [3], Estefanía Martínez [3], Sara Herrera [3], Stiven López [3], Ana BJ Sobrido [4], Cristina I. Castro[3]

[1] Semillero de Termofluidos y Conversión de la Energía, Ingeniería Química, Escuela de Ingenierías, Universidad Pontificia Bolivariana, Medellín (Colombia)
[2] Grupo de Energía y Termodinámica, Ingeniería Química, Escuela de Ingenierías, Universidad Pontificia Bolivariana, Medellín (Colombia)
[3] Grupo de Investigación sobre Nuevos Materiales, Ingeniería en Nanotecnología, Escuela de Ingenierías, Universidad Pontificia Bolivariana, Medellín (Colombia)
[4] School of Engineering and Materials Science, Queen Mary University of London, London, United Kingdom

*zulamita.zapata@upb.edu.co

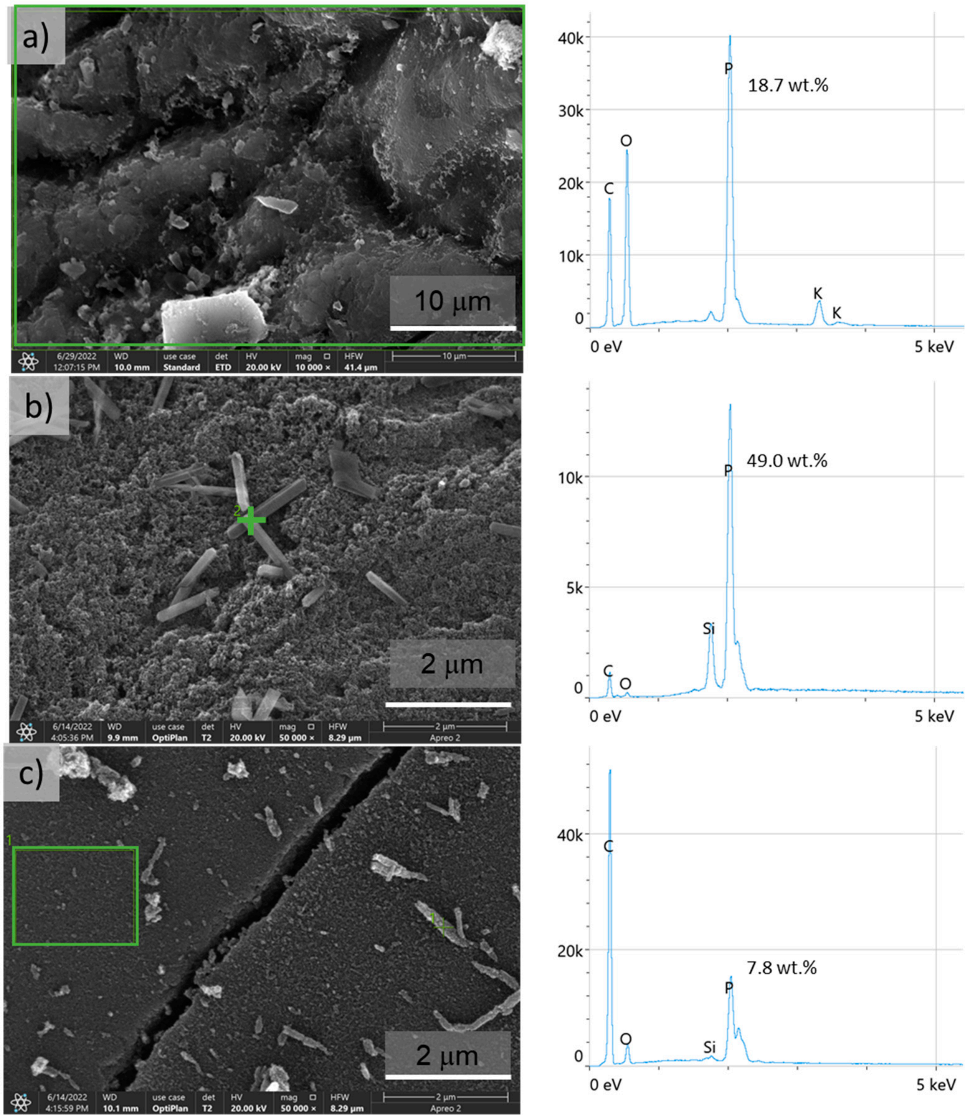

Figure S1. SEM images and EDX spectra of BNCA-P at 500 °C X10000 (**a**) and 600 °C X50000 (**b** and **c**).

Table S1. Binding energies of functional groups of XPS high-resolution spectra C_{1s}, O_{1s}, and P_{2p}.

C_{1s} [1, 2]		O_{1s} [1, 3]		P_{2p} [3]	
B.E. (eV)	Functional group	B.E. (eV)	Functional group	B.E. (eV)	Functional group
284.6	C=C	530.5	Quinone C=O	132.2	C_3-PO
285.3	C-C sp^3 aliphatic	531.3	O=C + O=P	133.5	C-PO_3 o C_2-PO_2
287.5	R-OH and C-O-C and/or C-O-P	532.4	O-C + COH + C-O-C + P-O	134.5	C-O-P, $(CO)_3PO$, $(CO)_2PO_2$ and $(CO)PO_3$
286.3	C=O + >C=O	533.3	C-OH and/or P-O- P		
289.3	-C(O)-O-C	534.5	Oxygen chemisorb and H_2O		
291.6	π–π*				

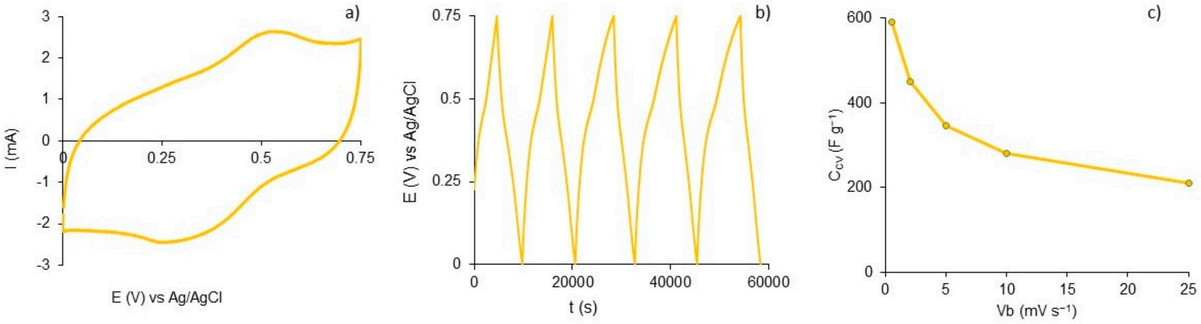

Figure S2. (**a**) Cyclic voltammetry curves at 0.5 mV s^{-1}, (**b**) Charge/Discharges curves at 0.125 A g^{-1}, and (**c**) Capacitance as a function at different scan rates for K700 sample.

References

1. Puziy AM, Poddubnaya OI, Ziatdinov AM (2006) On the chemical structure of phosphorus compounds in phosphoric acid-activated carbon. Appl Surf Sci 252:8036–8038
2. Li W, Wang J (2016) Significant enhancement of the cycling performance and rate capability of the P/C composite via chemical bonding (P-C). J Mater Chem A 4:505–511
3. Liu Y, Li K, Liu Y, et al (2015) The high-performance and mechanism of P-doped activated carbon as a catalyst for air-cathode microbial fuel cells. J Mater Chem A 3:21149–21158
